# Unusual mtDNA Control Region Length Heteroplasmy in the COS-7 Cell Line

**DOI:** 10.3390/genes11060607

**Published:** 2020-05-30

**Authors:** Nataliya Kozhukhar, Sunil Mitta, Mikhail F. Alexeyev

**Affiliations:** Department of Physiology and Cell Biology, University of South Alabama, Mobile, AL 36688, USA; nkozhukhar@southalabama.edu (N.K.); sm1921@jagmail.southalabama.edu (S.M.)

**Keywords:** mitochondrial DNA, control region, variable number tandem repeats, *Chlorocebus aethiops*, mtDNA length heteroplasmy

## Abstract

The COS-7 cell line is a workhorse of virology research. To expand this cell line’s utility and to enable studies on mitochondrial DNA (mtDNA) transcription and replication, we determined the complete nucleotide sequence of its mitochondrial genome by Sanger sequencing. In contrast to other available mtDNA sequences from *Chlorocebus aethiops*, the mtDNA of the COS-7 cell line was found to contain a variable number of perfect copies of a 108 bp unit tandemly repeated in the control region. We established that COS-7 cells are heteroplasmic with at least two variants being present: with four and five repeat units. The analysis of the mitochondrial genome sequences from other primates revealed that tandem repeats are absent from examined mtDNA control regions of humans and great apes, but appear in lower primates, where they are present in a homoplasmic state. To our knowledge, this is the first report of mtDNA length heteroplasmy in primates.

## 1. Introduction

The COS-7 cell line (CV1 in origin, SV40-transformed) was derived by Gluzman et al. in 1981 by transforming the African green monkey kidney epithelial cell line CV1 (derived ca. 1963) with SV40 virus carrying a defect in the origin of replication [1,2]. The resulting cell line retains complete permissiveness for the lytic growth of SV40, supports the replication of tsA209 virus at 40 degrees C and supports the replication of pure populations of SV40 mutants with deletions in the early region, which is consistent with the integration of the early region of SV40, including wild-type t-antigens. It was originally developed for the propagation of pure populations of recombinant SV40 viruses; however, its uses also includes the propagation of polyomavirus and rotavirus [3,4], as well as research on ion channels [5], apoptosis and autophagy [6], immunology [7], cell biology [8], vitamin [9], hormone [10] and cell signaling [11,12].

Since kidney epithelium, from which CV1 cells are derived, is non-steroidogenic, COS-7 cells were extensively used as a transfection host in steroid research [13,14]. However, recent studies indicate that these cells express steroidogenic enzymes and metabolize steroids [15].

Interestingly, despite the relative evolutionary proximity of humans and African green monkeys, human cells are apparently unable to replicate COS-7 mitochondrial DNA (mtDNA) [16]. This makes COS-7 cells an excellent experimental system to study the molecular composition and operation of the interspecies barrier to mtDNA replication. Such studies would require the availability of the full mtDNA sequence of the COS-7 cell line. At least three complete *Chlorocebus aethiops* mtDNA sequences are available from GenBank (KU682691.1, AY863426.1 and NC_007009.1). However, several primer pairs designed based on available *C. aethiops* mtDNA sequences repeatedly failed to amplify COS-7 mtDNA. This suggests that COS-7 mtDNA is at variance with published sequences, which prompted us to determine the full nucleotide sequence of the mitochondrial genome in this cell line. Unexpectedly, in the process, we discovered a 108 bp sequence that was tandemly repeated up to five times in the control region.

In human mtDNA, the control region is located between MT-TP and MT-TF, and has a length of approximately 1122 bp. While the functional significance of this region, which contains promoters for mtDNA transcription, mtDNA heavy chain replication and other regulatory elements, is often emphasized, this region also contains DNA stretches of no known functional significance, some of which are prone to accumulating mutations. Hypervariable regions HVR1 and HVR2 in human mtDNA and variable number tandem repeats (VNTRs) found in the mtDNA of other species are examples of such poorly conserved sequences. Variation in the number of repeat elements within VNTRs has been reported between species of the same genus [17], between populations of the same species [18,19], and even within individual organisms [20]. In the latter case, both the mtDNA length heterogeneity between tissues and the length heteroplasmy within tissues can be observed [20].

## 2. Materials and Methods

### 2.1. Cell Lines and Propagation

COS-7 cells were purchased from the American Type Culture Collection (ATCC CRL-1651) and propagated in a DMEM medium supplemented with 10% fetal bovine serum and 50 mg/mL gentamicin at 37 °C in a humidified atmosphere containing 5% CO_2_. CV1 and COS TS 1 cell lines were obtained from Biosciences Divisional Services, University of California-Berkeley, and cultivated under the same conditions, except the COS TS 1 cells which were grown at 33 °C. The derivation and cultivation of the human osteosarcoma 143B cells devoid of mtDNA (143B ρ^0^ cells) were described previously [21].

### 2.2. Recombinant DNA

Recombinant DNA procedures were performed as described elsewhere [22]. Briefly, unmodified PCR fragments generated with Platinum Superfi DNA polymerase (Thermo Fisher Scientific, Waltham, MA, USA) were gel-purified using the Qiaquick gel extraction kit (Qiagen, Germantown, MD, USA) and ligated into EcoRV-digested pBluescriptII SK+ vector in the presence of an EcoRV enzyme to prevent vector self-ligation. After the transformation of the ligation mix into *E. coli* GeneHogs (Thermo Fisher Scientific, Waltham, MA, USA), white colonies were picked on plates containing ampicillin (200 mg/mL) and X-gal (40 mg/mL), expanded overnight in TB medium, and used for plasmid DNA extraction (Qiaprep Spin miniprep kit, Qiagen, Germantown, MD, USA). After confirming the presence of the mtDNA insert by restriction digest with *Eco*RI and *Hind*III restriction enzymes, the plasmid DNA was used for sequencing or other analyses. Similarly, the PCR products containing tandem repeats (with short adjucent vector sequences) were released from the vector by digesting the recombinant plasmids with *EcoR*I and *Hind*III.

### 2.3. Template Preparation and Sanger Sequencing

The near-complete mitochondrial genome of the COS-7 cell line was amplified with long-range PCR using the GoTaq^®^ Long PCR Master Mix (Promega, Madison, WI, USA) and primers Agm15617F and Agm15617R (Supplementary Table S1) in 50 μL reactions using the following cycling parameters: initial denaturation at 95 °C for 2 min, followed by 45 cycles at 92 °C for 10 s and at 65 °C for 15 min, followed by a final extension for 10 min at 72 °C. The amplification with GoTaq G2 DNA polymerase (Promega, Madison, WI, USA) was performed with initial denaturation at 95 °C for 1 min, followed by 35 cycles at 94 °C for 10 s, at 55 °C for 15 s, and at 68 °C for 30 s followed by a final extension for 5 min at 68 °C. The amplification with Platinum Superfi DNA polymerase was performed using the initial denaturation at 98 °C for 30 s, followed by 35 cycles at 98 °C for 5 s, at 55 °C for 10 s and at 72 °C for 1 min followed by a final extension for 5 min at 72 °C. For sequencing, successful amplification was confirmed by agarose gel electrophoresis and the reactions were treated with *Escherichia coli* Exonuclease I and recombinant shrimp alkaline phosphatase (2 units and 0.2 units per 50 mL reaction, respectively) (Thermo Fisher Scientific, Waltham, MA, USA) for 30 min at 37 °C. The enzymes were inactivated by incubating the reaction mix for 15 min at 80 °C, and the resulting product was used without further purification as a template in sequencing reactions containing 1 mL of the template, 0.5 mL of BigDye v3.1 mastermix (Thermo Fisher Scientific, Waltham, MA, USA), 1.75 mL of 5x BigDye dilution buffer (Thermo Fisher Scientific, Waltham, MA, USA), 2 mL of 2 pMol/mL of the corresponding sequencing primer (Supplementary Table S1) and 4.75 μL of water. The sequencing reactions were cycled as follows: the initial denaturation 96 °C for 1 min followed by 45 cycles at 96 °C for 10 s, at 54 °C for 10 s and at 60 °C for 4 min. The sequencing products were purified by ethanol precipitation as recommended by the sequencing kit manufacturer, and the dry pellets were submitted for a capillary run to Functional Biosciences (Madison, WI, USA). The resulting traces were aligned using SeqManPro (DNAStar, Madison, WI, USA). The sequences of the mtDNA fragments cloned in pBluescriptII SK+ were determined in a similar fashion using amounts of template DNA and primers recommended by the sequencing kit manufacturer (Applied Biosystems, Waltham, MA, USA). Tandem repeats in mtDNA were identified using Tandem Repeat Finder [23].

### 2.4. Simultaneous Amplification of the nDNA and mtDNA

To resolve the potential contribution of the nuclear mitochondrial sequences to the observed apparent mtDNA length heteroplasmy, we designed primers for the amplification of a nuclear locus (GenBank NC_02657.1, Supplementary Table S1). These primers were tested on COS-7 genomic DNA, the PCR fragment was cloned, sequenced, and the sequence matched that of NC_02657.1. COS-7 genomic DNA was diluted to 1 ng/mL, and serial 3-fold dilutions of this DNA were amplified with GoTaq G2 green DNA polymerase 2x mastermix in 20 mL reactions that contained 1 mM of each primer (agmNUCf, agmNUCr, agmF33, and agmR_6, Supplementary Table S1).

## 3. Results and Discussion

By systematically checking different primer pairs designed against GenBank AY863426.1 for the amplification of the COS-7 mtDNA, we identified primers agmMitF10556 and agmMitR11565, which amplified a 1009 bp fragment (Supplementary Table S1). This fragment was cloned in a pBluescript II SK+ vector, and the sequences of inserts in three clones were determined by Sanger sequencing. The consensus sequence most closely matched *C. aethiops* KU682691.1 and was used to design primers for inverse PCR. Two primers, Agm15617F and Agm15617R, were chosen to amplify the 15,617 bp near complete mtDNA of COS-7. This PCR product was subjected to an initial round of sequencing with primers agmF1-agmF32 and agmR_1-agmR_29 (Supplementary Table S2). The gaps were closed by PCR-amplifying respective regions using sequencing primers and sequencing the resulting shorter fragments with additional primers (Supplementary Table S1). In cases where this approach did not result in the closing of the gap, the PCR fragments were cloned in pBluescriptII SK+ and sequenced as part of a plasmid.

**Figure 1:**
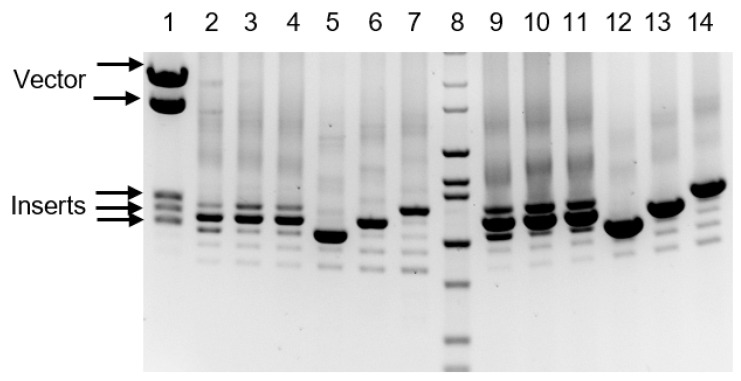
Analysis of the tandem repeats in COS-7 mitochondrial DNA (mtDNA). Lane 1: The three longest PCR products were cloned in the plasmid pBluescriptII SK+; the resulting recombinant plasmids were mixed and digested with restriction endonucleases flanking the site of insertion to release cloned fragments. Lanes 2–4: genomic DNA from the COS TS 1, COS-7 and the CV1 cell lines, respectively, amplified with primers agmF33 and agmR_1 with Platinum Superfi DNA polymerase. Lanes 9–11: the same samples amplified with GoTaqG2. Lanes 5–7: DNA from 143B ρ0 cells was spiked with plasmids containing PCR fragments with three, four or five 108 bp repeats, respectively, and amplified as in lanes 2–4. Lanes 12–14: DNA samples as in lanes 5–7 amplified with GoTaqG2. Lane 8, HiLo DNA ladder.

After attempts to close the gap around the control region using additional primers (Supplementary Table S1), and either a near-complete mtDNA PCR product or a shorter ~3.5 kbp PCR fragment encompassing the D-loop (generated with primers agmF33 and agmR_6), failed to produce a “clean” sequence, we attempted to clone an even shorter fragment generated with primers agmF33 and agmR_1 (Supplementary Table S1). Unexpectedly, the PCR reaction resulted in a ladder of fragments (Figure 1, lane 3) suggesting the length heterogeneity of the control region, or the presence of variably amplified repetitive sequence(s), or both. The three longest (and most prominent) PCR products were cloned in the EcoRV site of pBluescriptII SK+, and sequenced with vector-specific T3 and T7 primers. The sequencing revealed that the three major amplification products contained the same 108 bp sequence tandemly repeated three, four or five times. This outcome raised a question of whether multiple amplification products were an artifact of amplification. Indeed, the PCR amplification of tandem repeats can result in products with a reduced number of repeat units and under certain conditions can lead to an increase in the number of repeats [24,25].

Due to historical remoteness, DNA samples from the donor animal used to derive the CV1 cell line were not available for our studies. Therefore, we used agmF33 and agmR_6 primers to amplify the mtDNA control region in the COS TS 1 and CV1 cell lines with Platinum Superfi DNA polymerase (Thermo Fisher Scientific, Waltham, MA, USA). These PCR reactions also yielded a spectrum of products, which was identical to that generated in the COS-7 cell line (Figure 1, lanes 2 and 4, respectively). The CV1 cell line is parental to both the COS-7 and COS TS 1 cell lines, which were derived from CV1 at approximately the same time. Therefore, the most parsimonious explanation for these results is that these tandem repeats were already present in the CV1 cell line at the time of COS-7 and COS TS 1 derivation. However, we are unable to exclude the possibility that these repeats arose during or after establishing the culture of CV1. Nevertheless, the available evidence supports the existence of long tandem repeats in the control region of *Chlorocebus* (Table 1), whereas the induction of direct repeats in mtDNA by introducing the cells into culture has to date not been reported in the literature. Therefore, the most likely explanation is that the 108 bp repeats were present in the mtDNA of the animal at the time of establishing the CV1 cell culture.

The final mtDNA sequence of the COS-7 cell line (GenBank MN816163) is most similar to that of *C. aethiops* (GenBank KU682691.1), to which it is 95.8% identical. This is consistent with the documented derivation of the COS-7 cell line from *Cercopithecus aethiops* [1,2].

To address the question of whether the observed length heterogeneity of the control region is an artifact specific to Platinum Superfi DNA polymerase, we amplified the same DNA samples with GoTaq G2 DNA polymerase (Promega, Madison, WI, USA). This amplification resulted in an identical spectrum of PCR products, although their relative intensity may have slightly changed (Figure 1, lanes 9–11). Therefore, the control region length heterogeneity is not likely to be a polymerase-specific artifact.

To further address the issue of potential PCR artifacts, we established reference patterns of control region amplification using cloned PCR fragments of a defined length. To that end, we emulated the total cellular DNA from the COS-7 cell line by mixing the genomic DNA from a human osteosarcoma cell line 143B ρ^0^ that lacked mtDNA with plasmid DNA that contained cloned *C. aethiops* control regions with three, four or five 108 bp repeats in a molar ratio 1:20 (mtDNA in COS-7 cells is present at approximately 20 copies per cell, results not shown). The amplification of these DNA samples with either Platinum Superfi or GoTaq G2 DNA polymerases resulted in essentially identical banding patterns (Figure 1, lines 5–7 and 12–14, respectively). Three conclusions can be drawn from this experiment. The first is that PCR amplification can indeed generate a heterogenous mix of products from a homogenous template containing a repetitive sequence. In our case, PCR reactions generated mixes of fragments containing a number of 108 bp elements that ranged from one to the number of repeats contained in the plasmid used to spike the human DNA sample. The second is that the PCR product containing the correct number of repeats is by far the most predominant one, and that no discrete products with an increased number of repeats were observed, regardless of the DNA polymerase used for amplification. This may have implications for the interpretation of complex patterns of mtDNA amplification. The third conclusion is that a comparison of the spectra of PCR products generated of the total DNA isolated from the cell lines (Figure 1, lanes 2–4 and 9–11) with those generated on chromosomal/plasmid DNA mixtures (Figure 1, lanes 5–7 and 12–14) clearly indicate the mtDNA control region length heterogeneity in all three cell lines studied. Indeed, among PCR products obtained using total cellular DNA as a template, the band corresponding to a product with five 108 bp repeats is weaker than that corresponding to a product with four repeats. This pattern of amplification cannot be reproduced by mixes containing plasmid DNAs with cloned fragments containing either four or five repeats and suggests the presence of at least two mtDNA species in cells.

It is conceivable that the co-amplification of the nuclear mitochondrial sequences (NUMTs) containing an alternative number of repeats can manifest itself as mtDNA length heteroplasmy. However, three considerations make this possibility unlikely:

1. mtDNA in COS-7 cells is present at >20 copies per cell (our unpublished observations). Assuming that NUMTs and mtDNA are amplified with the same efficiency, the abundance of NUMTs in the final PCR product is expected to be ~5%. As we indicated earlier, we were unable to sequence the near genomic length PCR product (15,617 bp) through this repeat region because of a mixed sequence. However, it is known that Sanger sequencing (implemented without special accommodations as in our case) can only detect a variant sequence if its abundance is >20% [26]. Therefore, we should not have noticed interference from NUMTs, and sequencing difficulties were likely due to a bona fide mtDNA sequence length heteroplasmy.
2. Conversely, all near genomic length NUMTs that we are aware of differ in sequence from the resident mtDNA (and ours would be clearly diverged by having an alternate number of repeats). Therefore, if for some reason NUMTs are preferentially amplified so that their abundance increased to >20% to the level detectable by Sanger sequencing, we should have also detected heteroplasmy at other loci, which was not the case.
3. Finally, for a 15,617 bp NUMT to be amplifiable, at least three conditions should be met: (a) the NUMT should have a near-genomic length (these are outnumbered by shorter NUMTs in all known genomes. Shorter NUMTs can not be amplified with our long-range primers), (b) our primers should have sufficient homology to NUMTs, and (c) the breaking point for NUMT insertion should have occurred within the short 777 bp region between our primers for long-range amplification, which is unlikely. Collectively, these considerations led us to believe that mtDNA length heteroplasmy in the COS-7 cell line did not result from NUMT contribution.

To experimentally address this NUMT concern, we examined the relative amplification of the nuclear DNA (nDNA) vs. the hyputative length-polymorphic region of mtDNA. The anticipation was that if NUMTs contribute a specific length isoform/fragment length, which is not present in mtDNA, then the amplification of this fragment in serial dilutions of genomic DNA should cease when an nDNA fragment ceases to amplify. To ensure the robustness of this test, the nDNA fragment (570 bp) was approximately half the size of the mtDNA fragments containing four or five repeats (1147 and 1255 bp, respectively), which skewed amplification in its favor (shorter fragments tend to amplify more efficiently). Nevertheless, five- and four- repeat mtDNA fragments continued to amplify through at least four serial dilutions after the nDNA ceased to amplify (Supplementary Figure S1). This observation strongly suggests that NUMTs are not contributory to COS-7 mtDNA length polymorphism.

A brief survey of mtDNA sequences in primates revealed that tandem repeats longer than three nucleotides are absent from the control regions of the mitochondrial genomes of humans and great apes. They appear in the mtDNA of lesser apes, monkeys, prosimians and distantly related *Dermoptera* (Table 1). Intra- and interspecies variability in the tandem repeat presence, the repeat unit length, and the number of units in the repeats are also obvious. However, to our knowledge, the heteroplasmy in the lengths of tandem repeats in primates has not previously been reported. Moreover, the repeat region in the COS-7 cells was the longest among those examined.

This study also has methodological implications. Here, we established that the PCR amplification of mtDNA regions containing long direct repeats can generate products with a reduced number of repeat elements. PCR is a standard approach for the template preparation of mtDNA sequencing. Therefore, there is potential for PCR-mediated artifacts to be interpreted as mtDNA length heteroplasmy. Our survey of the literature suggests that this possibility is not always considered. Here, we addressed the issue by cloning PCR fragments with various numbers of repeat units and using the resulting recombinant plasmids to reproduce the amplification pattern observed with the samples of total cellular DNA. This study also illustrates some limitations of short read next-generation sequencing approaches for mtDNA sequencing. Indeed, Illumina 50 or 75 bp reads would have missed the VNTRs in the COS-7 control region altogether, whereas longer 150 bp and 300 bp reads would have had difficulty resolving the exact number of repeat units or detecting mtDNA length polymorphism.

## 4. Conclusions

In conclusion, we would like to note that the observations of inter- and intraspecies mtDNA length heterogeneity in primates and other metazoans have implications for our understanding of mtDNA biology. This field is currently heavily dominated by concepts derived from studies of human mtDNA. Human mtDNA manifests a minimal length heterogeneity of the control region, which has biased our views. Hypervariable regions HVR1 and HVR2 in human mtDNA and the VNTRs found in the mtDNA control regions of other species clearly indicate the plasticity of this region in terms of both sequence identity and sequence length. Therefore, a balanced view of the mtDNA control region as an element that harbors not only highly conserved, functionally important sequences, but also very plastic ones, appears optimal.

## Figures and Tables

**Table 1:** Prevalence of the tandem repeats in control regions of the select primate mitochondrial genomes *.

Species	Accession#	Unit Length	Number of Units	Repeat Length	% Unit Identity	Group
*Homo sapiens*	NC_012920.1	0	0	0	0	Humans
*Pan paniscus*	HM015213.1	0	0	0	0	Great apes
	GU189677.1	0	0	0	0	
	GU189676.1	0	0	0	0	
	GU189675.1	0	0	0	0	
	GU189674.1	0	0	0	0	
	JF727231.3	0	0	0	0	
	JF727228.2	0	0	0	0	
*Pan troglodytes*	JF727179.2	0	0	0	0	Great apes
	JF727176.2	0	0	0	0	
	JF727166.2	0	0	0	0	
	JF727173.1	0	0	0	0	
	EU095335.1	0	0	0	0	
	NC_001643.1	0	0	0	0	
*Gorilla gorilla*	NC_001645.1	0	0	0	0	Great apes
	KF914214.1	0	0	0	0	
	NC_011120.1	0	0	0	0	
*Pongo pygmaeus*	NC_001646.1	0	0	0	0	Great apes
	KU353723.1	0	0	0	0	
	D38115.1	0	0	0	0	
	NC_002083.1	0	0	0	0	
*Hoolock leuconedys*	KY250074.1	36	1.9	68	93	Lesser apes
	NC_033885.1	0	0	0	0	
	NC_033884.1	0	0	0	0	
	NC_033883.1	0	0	0	0	
	NC_033882.1	0	0	0	0	
*Hylobates lar*	NC_002082.1	0	0	0	0	Lesser apes
	HQ622775.1	0	0	0	0	
	NC_002082.1	0	0	0	0	
*Hoolock hoolock*	NC_033885.1	0	0	0	0	Lesser apes
*Chlorocebus aethiops*	MN816163	108	5	540	100	Old world
	NC_007009.1	0	0	0	0	
	KU682691.1	0	0	0	0	
*Chlorocebus pygerythrus*	KU682698.1	0	0	0	0	Old world
	KU682694.1	0	0	0	0	
	KU682696.1	0	0	0	0	
	EF597501.1	0	0	0	0	
	EF597500.1	0	0	0	0	
	NC_009747.1	0	0	0	0	
*Chlorocebus cynosuros*	KM262190.1	0	0	0	0	Old world
	JQ256915.1	0	0	0	0	
	KU682693.1	0	0	0	0	Old world
	NC_024933.1	0	0	0	0	
*Chlorocebus sabaeus*	KU682697.1	122	3.1	378	100	Old world
	NC_008066.1	0	0	0	0	
	DQ069713.1	0	0	0	0	
	EF597503.1	0	0	0	0	
*Chlorocebus djamdjamensis*	KU682695.1	0	0	0	0	Old world
*Chlorocebus tantalus*	KU682699.1	130	2.1	273	99	
	EF597502.1	0	0	0	0	
	KU682700.1	0	0	0	0	
	NC_009748.1	0	0	0	0	
*Allenopithecus nigroviridis*	NC_023965.1	0	0	0	0	Old world
	KJ434962.1	0	0	0	0	
	JQ256993.1	0	0	0	0	
*Alouatta seniculus*	HQ644333.1	10	3.7	37	100	New world
	NC_027825.1	10	3.7	37	100	
*Saimiri oerstedii oerstedii*	HQ644337.1	0	0	0	0	New world
*Tarsius dentatus*	NC_024052.1	35	9.8	343	99	Tarsiers
*Indiri Indiri*	NC_026095.1	20	13.4	268	91	Lemuriformes
	KJ944237.1	20	13.4	268	91	
	KJ944258.1	24	12.8	307	96	
	KJ944231.1	22	14.6	321	93	
	KJ944231.1	28	7.9	221	90	
	KJ944198.1	44	6.7	294	91	
*Loris lydekkerianus*	NC_021955.1	14	18.9	264	95	Lemuriformes
*Galago senegalensis*	NC_012761.1	14	18.6	260	98	Lemuriformes
*Galeopterus variegatus*	NC_004031.1	40	4.5	180	98	Dermoptera

* When more than one repeat region is present in the control region, the longest is reported.

## Supplementary Materials

The following are available online at https://www.mdpi.com/2073-4425/11/6/607/s1, Table S1: Oligonucleotides used in this study. Table S2: Sequencing primers. Figure S1: Relative amplification of the mtDNA tandem repeats region vs. nDNA.
