# Supplementary material for: Unusual mtDNA Control Region Length Heteroplasmy in the COS-7 Cell Line
Source: Genes (Basel). 2020 May 30;11(6):607. doi: 10.3390/genes11060607 (PMC7348793; doi:10.3390/genes11060607)
Supplement: Supplementary file 1 [file genes-11-00607-s001.pdf]

Table S1. Oligonucleotides used to amplify mtDNA templates for sequencing.

Name	Sequence	Position of the 5′-end on COS-7 mtDNA	Fragment length, bp
Agm15617F	gccctctactccctccacatatt	11387	~15617*
Agm15617R	ttgaggcgttctgcttggttac	10608	
agmMitF10556	cacccttattcccaccctaatc	10549	1009
agmMitR11565	acaggaggtgaaccctagaa	11557	
agmF33	ctaactcatgtcgttattgatc	1,079	3746
agmR_6	gggttaggtatgggaagggg	3005	
agmF33	ctaactcatgtcgttattgatc	16079	1255
agmR_1	gggctaagcatagtggggta	514	
agmNUCf	gtcagagagaagagggctagaa	n/a	570
agmNUCr	tcttgtgctagatgacaccaac	n/a	

*Based on GenBank KU682691.1
n/a, not applicable

Table S2. Sequencing primers.

Name	Sequence	Position of the 5′-end on COS-7 mtDNA
agmF1	ccccaaagcaagacactgaa	17
agmF2	agctaaaccccacctaaactgt	387
agmF3	agagtgcttaattgaacgaggc	862
agmF4	tgggaagatttacgggtagagg	1382
agmF5	aggaactcggcaaaactaaacc	1879
agmF6	ccatatcaacaatagagtttacgacc	2374
agmF7	tacggactactacaacccttcg	2851
agmF8	gcccattcgccctattctttat	3377
agmF9	tcacacccttcccgtactaatt	3868
agmF10	gaggcctaaaccagacacaatt	4389
agmF11	tcaccatcatttctaccctcct	4872
agmF12	cacaaagacatcggaaccctat	5354
agmF13	ccccattgtttgtctgatctgt	5862
agmF14	accggtattatcttagcaaactca	6380
agmF15	aaccggtttcaagccaatccta	6890
agmF16	cttaaacccaggagaccttcga	7384
agmF17	actgctaaacatatattactatcaaccc	7864
agmF18	gtcatccaaccagtagccct	8392
agmF19	accttcgatatggcatagtcct	8857
agmF20	tctctatttactgatgagggtcct	9385
agmF21	tcaccatactaaccaaatgtcacc	9872
agmF22	ctcttctgacactaaccgcttg	10369
agmF23	cgccgcagtactcttaaaacta	10870

agmF24	ctaatcacagccctctactccc	11378
agmF25	gccttcattactagtcttcttcca	11882
agmF26	ctaaattccaaccccaaacccc	12365
agmF27	cctcacctcaacttccctactt	12856
agmF28	tgccacacctaaacctactcat	13365
agmF28.5	taaccaacaacacacttaacg	14050
agmF29	gacgtaaaccatggctgaatca	14376
agmF30	taacactactttcacccgacct	14887
agmF31	agaaagcacttgacttcaccac	15379
agmF32	tgtatccggcatctggttctta	15856
agmF33	ctaactcatgtcgttattgatc	16079
agmF34	gccaaagcttatattttatc	16276
agmF6.5	ttaacaaagcaccacaaatc	2609
agmR0.5	gggaggcttgcatgtgtaat	129
agmR_1	gggctaagcatagtggggta	514
agmR_2	tatccaagtgcaccttccgg	1015
agmR_3	acaaggggattaagtggttctg	1497
agmR_4	ctttgcacggtcagggtac	2003
agmR_5	attgctccggtctgaactca	2499
agmR5.5	agaagaggggttgaacctct	2717
agmR_6	gggttaggtatgggaagggg	3005
agmR_7	aggaggagtgttttggtggt	3521
agmR_8	tgtttatttctaggcctgctcag	3995
agmR_9	gggttgtgatggttgggttg	4493
agmR_10	tgcagtccttatgtgtttcaga	5012
agmR10.5	ttggcctagttcagctcgaa	5449
agmR_11	aggtgttggtataggatagggtc	6021
agmR11.5	tgggcgacaacatagtacgt	6447
agmR_12	tagttgaactgggtgggcc	7024
agmR_13	tgtggggatagttcatgagtgt	7504
agmR_14	gggattgtggcaggttgatt	8009
agmR_15	agatcagtgaagtagcgggg	8521
agmR15.5	tccgggtactgcatctgttt	7534
agmR15.7	acttctaagggatttaacggggt	9010
agmR_16	aagggtgttgattgttagagct	9512
agmR_17	ccatgagggcgattgtgatg	10029
agmR_18	tctgtggccatgaatgttaga	10518
agmR_19	cgtaggcagattgagcttgt	11010
agmR_20	tggggttaagggatagaagga	11531
agmR_21	gcgattggggtgaatattacgg	12033
agmR_22	agagtggagtagggctgaga	12502
agmR_23	gaggtggcgatgagagtgat	13005
agmR_24	tgagaaaggagagggagtatagc	13532
agmR_25	agccttctcctatttacgggg	14025
agmR_26	taggagaaggaatgagccgt	14492

agmR27.5	gatatctttgattgtgtagtag	14849
agmR27.4	cctaggatatctttgattgtg	14854
agmR_27	tcatgttggctagcagtaatgc	15529
agmR_28	ttgcgtgcagaccagagata	16018
agmR_29	gtaggtggtgtctttggggt	16801

Supplementary Figure S1.

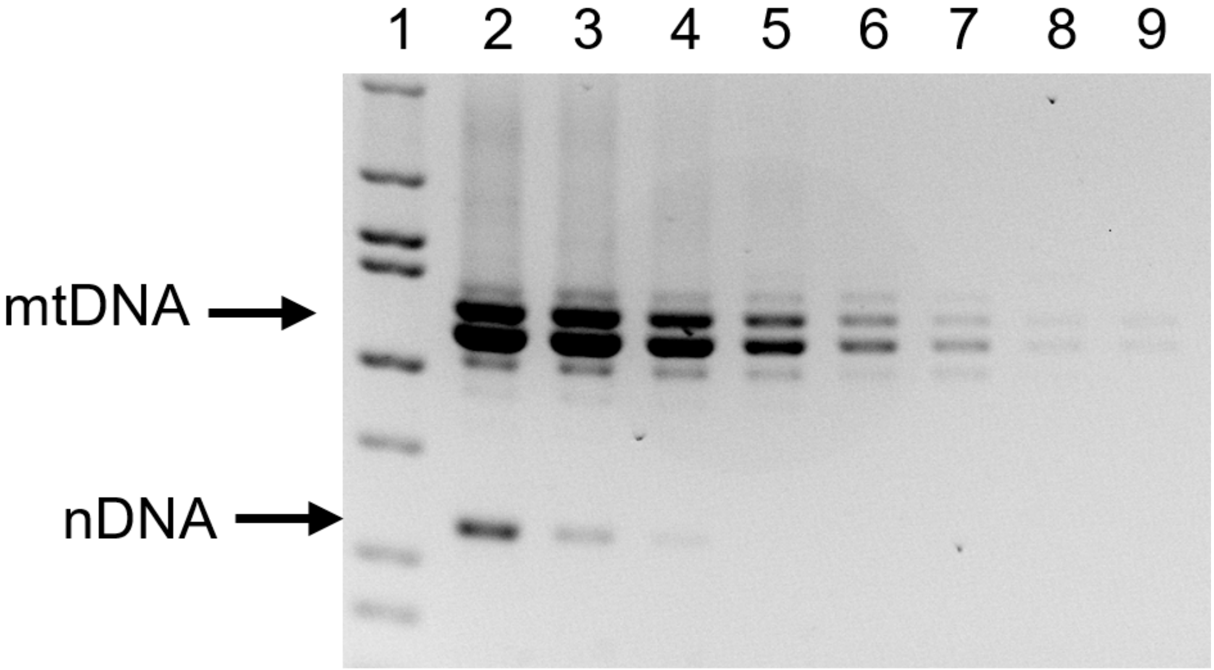

Figure S1. Relative amplification of the mtDNA tandem repeats region vs. nDNA. Lanes: 1. HiLo DNA ladder. Lanes 2-9, serial 3x dilutions of COS-7 genomic DNA were amplified with primers agmNUCf, agmNUCr, agmF33, and agmR_6 with products of amplification of 1 ng of DNA present in Lane 2.
